# Supplementary material for: Anemia status and its determinants among reproductive-age women in Tanzania: A multi-level analysis of Tanzanian demographic and health survey data
Source: PLoS One. 2024 Nov 18;19(11):e0311105. doi: 10.1371/journal.pone.0311105 (PMC11573225; doi:10.1371/journal.pone.0311105)
Supplement: Attachment — Submitted filename: Response to Reviewers.docx [file pone.0311105.s001.docx]

**Response to reviewers**

**May 30, 2024**

To: Dr Miquel Vall-llosera Camps (Staff Editor, PLOS ONE)

**PONE-D-24-03494: Anemia Status and its Determinants among Reproductive-age Women in Tanzania: A Multi-level analysis of Tanzanian Demographic and Health Survey Data**

Dear Academic Editor,

Thank you for considering our manuscript and for arranging for it to be reviewed by reviewers. We have tried to address your comments and the comments / suggestions from the reviewers. We have highlighted the changes within the manuscript please find for your kind consideration the following: In the Response to Reviewers, we copy each of the *comments* / *suggestions* and provide the **RESPONSE** hereunder. We also provide a marked-up copy of the manuscript that highlights changes made to the original version and this is uploaded as a separate file labeled “*Revised Manuscript with Track Changes*”. Finally, we provide an unmarked version of the revised manuscript without tracked changes and this is uploaded as a separate file labeled *'Manuscript*'.

We have been carefully through the peer review and have revised our paper accordingly. We feel that the paper is much improved as a result of this peer review process, and thank you for taking it to this stage. We hope we have satisfactorily addressed all the comments and hope that our paper may now be suitable for publication in your journal. We hold ourselves at your entire disposition for any further information or other changes you might require.

Sincerely yours!

Gizachew Ambaw	Kassie	on behalf of the co-authors

**Point by point response to reviewers**

**Editorial comment**: Please revise the manuscript to address all the reviewer's comments in a point-by-point response in order to ensure it is meeting the journal's publication criteria. Please note that the revised manuscript will need to undergo further review, we thus cannot at this point anticipate the outcome of the evaluation process. Please include the following items when submitting your revised manuscript: A rebuttal letter that responds to each point raised by the academic editor and reviewer(s). You should upload this letter as a separate file labeled 'Response to Reviewers'. A marked-up copy of your manuscript that highlights changes made to the original version. You should upload this as a separate file labeled 'Revised Manuscript with Track Changes'. An unmarked version of your revised paper without tracked changes. You should upload this as a separate file labeled 'Manuscript'.

**Response:** Dear academic editor, we would like to thank you for your time and effort in facilitating our manuscript revision. In the Response to Reviewers, we copy each comment and provide the response hereunder. We also provide a marked-up copy of the manuscript that indicates tracked changes made to the original version, and this is uploaded as a separate file labeled “Revised Manuscript with Track Changes” and an unmarked version of our revised paper without tracked changes uploaded as a separate file labeled '*Manuscript*'.

**Editorial comment 2:** **Journal Requirements**: When submitting your revision, we need you to address these additional requirements. Please ensure that your manuscript meets PLOS ONE's style requirements, including those for file naming. We noticed you have some minor occurrence of overlapping text with the following previous publication(s), which needs to be addressed: <https://doi.org/10.1371/journal.pone.0296451> In your revision ensure you cite all your sources (including your own works), and quote or rephrase any duplicated text outside the methods section. Further consideration is dependent on these concerns being addressed.

**Response:** Thank you for your comments and feedback, we have carefully reviewed and addressed all of your concerns. We have made sure that our manuscript meets PLOS ONE's style requirements, including file naming. Additionally, we have carefully checked the manuscript for any overlapping text with the previous publication(s) you mentioned and have addressed them. **Page #8, line #1-38**

**Reviewers' comments:**

Review Comments to the Author

**Reviewer #1:**

**General comment:** This study aimed to assess the burden of anemia and its determinants among reproductive aged women using the Tanzanian Demographic and Health Survey data. I made few comments that can improve the readability and quality of the manuscript mentioned below:

**Response:** Dear reviewer, we would like to express our gratitude for the time you have taken to insightful and constructive comments on our manuscript, and we appreciate the effort you have put in to review our paper. We have carefully considered all your comments and suggestions. **Page #2-28**

**Comment 1:** The background section needs to be improved, specifically the rationale of the study, though author tried to make the rationale clear.

**Response:** Thank you for your feedback. We acknowledge that the background section, particularly the rationale of the study, needs improvement. We have revised and provided a clearer and more compelling context for our research. **Page #3-5**

**Comment** **2**: Line 111-120: You need citation for classification of anemia. Just mentioning anemia level classification from the previous classification is not sufficient. Why you don’t follow the WHO criteria? Additionally, you need to explain what types of anemia you measured (like iron deficiency anemia)? See<https://www.cochranelibrary.com/cdsr/doi/10.1002/14651858.CD013092.pub2/full>.

**Response:** Thank you for your valuable comment. We now provide a citation for the classification of anemia. Specifically, we have incorporated the World Health Organization (WHO) criteria to enhance the clarity of our anemia classification. Regarding t**ypes of Anemia** we have included an explanation of the types of anemia measured in the revised version of our manuscript. **Page #7, line #149-154**

**Comment 3**: Line 121-133: You need further explanation and citation for several independent variables how you categorized them (e.g., BMI and so on).

**Response:** Thank you for your recommendation. We have further explained our variable measurements and provided citations for each operation definition in the revised manuscript. **Page #8-9, line #172-188**

**Comment 4**: Line 143: You mentioned that “logistic regression model was employed in our study since the anemia prevalence was high….”. How did you define high prevalence requires further explanation.

**Response:** Thank you for your feedback. We apologize for the editorial error. We have rephrased it in the revised version of our manuscripts. **Page #9, line #198**

**Comment** **5**: Could you please add a sampling framework of the study?

**Response: Thank you for your comment. We have taken your feedback into consideration and have now included a more detailed description of the sampling framework in the methodology section of the paper. Additionally, we have created a diagrammatic representation of the sampling process to provide a clearer understanding of how we selected our study participants. We hope that these additions will enhance the comprehensibility of our research and will address your concerns regarding the sampling framework. Thank you for your valuable feedback and suggestions for improving our work. Page #6-7, line #125-145**

**Comment** **6**: What did you mean no education? Explain in your method section.

**Response:** In our study, when we mentioned “no education,” we were referring to individuals who did not complete any formal education. We apologize for any confusion and appreciate the opportunity to clarify this point. In the revised method section, we have elaborated on this definition to ensure transparency and accuracy.

**Comment** **7**: Line 214-218 seems to be redundant as you explained it in the method section. Drop it.

**Response:** Thank you for your insightful comment. We appreciate your attention to detail. Based on your suggestion, we have removed it from the manuscript as they were redundant with the information already provided in the method section. **Page #15, line #267-271**

**Comment** **8**: Line 222: What is the meaning of significant association with anemia?

**Response:** Thank you for your question for clarification. The term “significant association” in our study refers to a statistically meaningful relationship between two variables. Specifically, when we say that there is a significant association with anemia, we mean that the presence of anemia is related to another factor (such as a risk factor, exposure, or outcome) in a way that is unlikely to occur by chance alone. **Page #15, line #275**

**Comment** **9**: Line 224-240: You mentioned that “…. Higher odds of a higher level of anemia….” which is completely inappropriate explanation. Also, you reported multivariate results in percentage and in times, please follow one style.

**Response:** Dear reviewer, we apologize for any confusion. In the revised manuscript, we have revised the interpretation to avoid ambiguity. Additionally, we have ensured consistency in reporting multivariate results by using a single style (either percentages or times). However, it is important to note that the interpretation of the odds ratio depends on whether the value is greater than 1 or less than 1. Therefore, it is crucial to consider the direction of the odds ratio when interpreting the results of a study. Thank you for bringing this important point to our attention. **Page #15-16, line #277-296**

**Comment** **10**: In discussion section, it is suggested to avoid quantitative results with Cis that you reported in result section. Please revise it.

**Response:** Dear reviewer, thank you for you insightful comments. We have removed the confidence interval based on your suggestion. We have been mentioned it in the discussion section for comparison purpose our result with other similar findings. **Page #18, line #308-310**

**Comment** **11**: Please provide a clear script of STATA code (do file) as an appendix.

**Response:** We appreciate the reviewer’s feedback. In response, we have uploaded comprehensive general STATA commands and the data set as “other” in the submission system manuscript.

**Reviewer #2:**

**General comment:** This is an interesting paper and utilized most recent data of Tanzanian DHS to assess anemia status and its determinants among reproductive-age women.

**Response: Response:** Dear reviewer, thank you for devoting your time to review our manuscript and we appreciate your deep insight of the manuscript. We have carefully considered all your comments and suggestions.

Methodology
**Comment 1:** In the study variables and measurements section you showed how the dependent variable was measured but it was not cited.

**Response:** Thank you for your feedback. We apologize for the oversight. In the revised manuscript, we have appropriately cited the references related to the measurement of the dependent variable in the study variables and measurements section. **Page #8-9, line #172-188**

**Comment 2:** The prevalence of anemia among women of reproductive age in Tanzania was reported 42.02%. Considering the cross-sectional nature of the data and the large prevalence of the outcome variable, odds ratio might overestimate the association between the dependent and independent variables. Therefore in my view multilevel Poisson regression analysis with robust variance would be preferable.

**Response:** Thank you for your valuable feedback regarding the statistical method used in our study. We appreciate your concern about the potential for odds ratios to overestimate the association between the dependent and independent variables. However, we carefully considered the characteristics of our dataset, research question, and study design and determined that odds ratios were the most appropriate statistical method to achieve our research objectives. Our study has a large sample size, which mitigates the influence of high prevalence of anemia among Tanzanian women of reproductive age while ensuring the precision of our odds ratio estimates. Moreover, our focus was on exploring relationships between anemia and various independent factors and not to forecast the prevalence rates of anemia. In this context, odds ratios are a robust measure that captures the strength and direction of these associations. Nonetheless, we appreciate your suggestion to use multilevel Poisson regression analysis with robust variance in future studies and will consider it for future research endeavors.
