# Supplementary material for: Anemia status and its determinants among reproductive-age women in Tanzania: A multi-level analysis of Tanzanian demographic and health survey data
Source: PLoS One. 2024 Nov 18;19(11):e0311105. doi: 10.1371/journal.pone.0311105 (PMC11573225; doi:10.1371/journal.pone.0311105)
Supplement: Attachment — Submitted filename: Response to Reviewer.docx [file pone.0311105.s002.docx]

**Response to reviewers**

**September 09, 2024**

To: Marly A. Cardoso, Ph.D. (Staff Editor, PLOS ONE)

**PONE-D-24-03494R1: Anemia Status and its Determinants among Reproductive-age Women in Tanzania: A Multi-level analysis of Tanzanian Demographic and Health Survey Data**

**Dear Academic Editor,**

Thank you for considering our manuscript and for arranging for it to be reviewed by reviewers. We have tried to address your comments and the comments / suggestions. We have highlighted the changes within the manuscript please find for your kind consideration the following: We also provide a marked-up copy of the manuscript that indicates tracked changes made to the original version, and this is uploaded as a separate file labeled “Revised Manuscript with Track Changes” and an unmarked version of our revised paper without tracked changes uploaded as a separate file labeled '*Manuscript*'.

We have been carefully through the peer review and have revised our paper accordingly. We feel that the paper is much improved as a result of this peer review process, and thank you for taking it to this stage. We hope we have satisfactorily addressed all the comments and hope that our paper may now be suitable for publication in your journal. We hold ourselves at your entire disposition for any further information or other changes you might require.

Sincerely yours!

Gizachew Ambaw	Kassie	on behalf of the co-authors

**Point by point response to reviewers**

**Editorial comment**: Thank you for submitting your manuscript to PLOS ONE. After careful consideration, we feel that it has merit but does not fully meet PLOS ONE’s publication criteria as it currently stands. Therefore, we invite you to submit a revised version of the manuscript that addresses the points raised during the review process. The manuscript was improved; however, significant scientific concerns remain. Please revise the manuscript to address all the reviewer's comments in a point-by-point response. In addition, a revision of the statistical analysis should be performed as recommended by the reviewer in the first revision round.  The authors’ reasons for not run a more appropriate statistical approach are not supported by the literature – the multilevel Poisson regression models are more appropriate for cross-sectional study design.

**Response:** Dear academic editor, we would like to thank you for your time and effort in facilitating our manuscript revision. Thank you for your feedback on our manuscript. We have tried to address all your and the reviewers’ comments in revised the manuscript. Specifically, we have clarified our methodology and revised the statistical analysis using multilevel Poisson regression models, as recommended. We believe these changes have significantly improved our manuscript. Thank you for considering our revised submission. **Page #17-20**

**Editorial comment 2:** The authors should revise the statistical analysis as recommended before by the reviewer as follows "The prevalence of anemia among women of reproductive age in Tanzania was reported 42.02%. Considering the cross-sectional nature of the data and the large prevalence of the outcome variable, odds ratio might overestimate the association between the dependent and independent variables. Therefore in my view multilevel Poisson regression analysis with robust variance would be preferable"

**Response:** Thank you for your comments and feedback, we have carefully reviewed and addressed all of your concerns. We have revised the statistical analysis as recommended by the reviewer. We have employed multilevel Poisson regression analysis with robust variance to better account for the cross-sectional nature of the data and the high prevalence of the outcome variable. **Page #2-25**

**Review Comments to the Author**

**Reviewer #3:**

**General comment:** Thank you for the opportunity to read this interesting manuscript that assessed the prevalence of anemia and its determinants among reproductive aged women using data from the Tanzanian Demographic and Health Survey. In my opinion, the manuscript is a good piece of work, well-structured and easy to follow and understand. The introduction section is well presented, provides a contextualization of the topic addressed and presents the justification for the study. The methods section is well detailed and can be replicated. However, I did not identify the inclusion and exclusion criteria for the study. Another observation is about the cutoff points adopted to assess the severity of anemia. According to the WHO document (ref 18), the cutoff points described in lines 140-142 are for pregnant women. Furthermore, the information present in lines 140-142 is different from the information present in lines 169-170. I suggest checking it out. In my opinion, the statistical analyses are sound and the results are robust. In the results section, I suggest presenting the results in the order they appear in the tables. Also, check the administrative zones associated with anemia (line 253). The central administrative zone is not associated with anemia according to Table 4. In the discussion section the authors explain the results and make relevant comparisons with previous studies, and the conclusions are well-stated.

**Response:** Thank you for the time and effort you have invested in reviewing our manuscript. We highly appreciate your valuable feedback and constructive criticisms on the manuscript. We have now revised the paper based on your comments and suggestions. **Page #2-24**

**Comment 1**: Line 64-65: I suggest including in the text the year in which this Tanzania Demographic and Health Survey was carried out.

**Response:** Thank you for your valuable feedback. We have included the year in which the Tanzania Demographic and Health Survey was conducted in the revised version of our manuscript. Specifically, we have noted that the survey was carried out in 2015/16. **Page #3, line #65**

**Comment 2:** Line 77: I suggest reviewing the text. Is there a period after “status”?

**Response**: Thank you for your suggestion. We have reviewed the text and addressed the issue regarding the period after “status.” The necessary correction has been made in the revised version of our manuscript. **Page #4, line #78**

**Comment 3:** Line 80: Include a period at the end of the sentence.

**Response:** Thank you for your comment and we have made correction accordingly on the revised version of the manuscript. **Page #4, line #81**

**Comment 4:** Line 140: I suggest presenting the acronym (Hgb) in full first.

**Response**: Thank you for pointing out it I have now presented the acronym “Hgb” in full as “hemoglobin” at its first occurrence in the document. This should enhance clarity for all readers. **Page #6, line #136**

**Comment** **5**: Line 141: I suggest correcting to “less than 7 g/dL” for severe anemia.

**Response**: Thank you for your feedback. I have accepted your comment and made the necessary corrections accordingly. **Page #7, line #141-148**

**Comment 6:** - Line 164: I think the topic “Operational definition”, when describing anemia, is a bit redundant. Additionally, I suggest that the BMI classification be in the “independent variables” topic.

**Response:** We appreciate your attention to detail. Based on your suggestion, we have removed it optional definition and merge with the independent variable section accordingly. **Page #18, line #169-182**

**Comment** **7**:-Lines 211 and 224: The values presented in % are different from the table. I suggest checking.

**Response:** Thank you for pointing this out. It was an editorial error we corrected it on the revised manuscript. **Page #11, line #234**

**Comment** 8- Table 4 (Model III): I suggest standardizing the number of “middle household wealth status”.

**Response:** Thank you very much for your valuable feedback and suggestion on our manuscript. All of our important findings have been presented in line with those reported in the TDHS results. **Page #18**

**Comment** 9**:** - Line 255-256: check the data on the “wealth index of the poorest families”. It is not in accordance with table 4.

**Response:** Dear reviewer, thank you for pointing out it. We have corrected in the revised manuscripts. **Page #18**

**Comment** 10: - Line 261: Please check the 95%CI of pregnancy. It is not in accordance with table 4.

**Response:** Thank you for your comment; we apologize for the incontinence we have corrected in the revised version. **Page #16, line #286**

**Comment** **11**:- Line 261: Please correct: “body mass index of <18.5 kg/m2”

**Response:** Dear reviewer, thank you for bringing that error to our attention. We apologize for the oversight. It was an editorial error made during paraphrasing and write-up of our results. We have made an extensive revision of our manuscript and corrected all editorial and typo errors. **Page #16, line #279**

**Comment** **12**: - Table 4: Why are the Southern Administrative Zone values in bold?

**Response:** Thank you again for bringing these errors. We accepted the comments and we have made the necessary correction in the revised version of our manuscript. **Page #19**
